# Efficacy and pharmacokinetic evaluation of a novel anti-malarial compound (NP046) in a mouse model

**DOI:** 10.1186/1475-2875-14-8

**Published:** 2015-01-06

**Authors:** Efrem T Abay, Jan H van der Westuizen, Kenneth J Swart, Liezl Gibhard, Nina Lawrence, Ntokozo Dambuza, Anke Wilhelm, Kendrekar Pravin, Lubbe Wiesner

**Affiliations:** Department of Medicine, Division of Clinical Pharmacology, University of Cape Town, Observatory, 7925 Cape Town, South Africa; PAREXEL® International Clinical Research Organization, Private Bag X09, Brandhof, 9324 Bloemfontein, South Africa; Department of Chemistry, University of the Free State, PO Box 339, Bloemfontein, 9300 South Africa; Research Development, University of the Free State, PO Box 339, Bloemfontein, 9300 South Africa

**Keywords:** Malaria, Drug development, Pharmacokinetics, *in vivo* efficacy

## Abstract

**Background:**

Even though malaria is a completely preventable and treatable disease, it remains a threat to human life and a burden to the global economy due to the emergence of multiple-drug resistant malaria parasites. According to the World Malaria Report 2013, in 2012 there were an estimated 207 million malaria cases and 627,000 deaths. Thus, the discovery and development of new, effective anti-malarial drugs are required. To achieve this goal, the Department of Chemistry at the University of the Free State has synthesized a number of novel amino-alkylated chalcones and analogues, which showed *in vitro* anti-malarial activity against both chloroquine-sensitive and chloroquine-resistant *Plasmodium falciparum* strains. The lead compound (NP046) was selected for a comprehensive pharmacokinetic (PK) and *in vivo* efficacy evaluation in a mouse model.

**Methods:**

*In vivo* efficacy: Water solutions of NP046 were administered orally at 50 and 10 mg/kg using oral gavage and IV at 5 and 1 mg/kg via the dorsal penile vein to *Plasmodium berghei* (ANKA strain) infected male C57BL/6 mice (n = 5), once a day for four days. Blood samples were collected *via* tail bleeding in tubes containing phosphate buffer saline (PBS) on day five to determine the % parasitaemia by flow cytometry.

*In vivo* PK: NP046 solutions in water were administered orally (50 and 10 mg/kg) and IV (5 mg/kg) to male C57BL/6 mice (n = 5). Blood samples were collected *via* tail bleeding into heparinized tubes and analysed using a validated LC-MS/MS assay. Data obtained from the concentration-time profile was evaluated using Summit PK software to determine the PK parameters of NP046.

**Results:**

NP046 inhibited parasite growth for the oral and IV groups. Better parasite growth inhibition was observed for the IV group. The PK evaluation of NP046 showed low oral bioavailability (3.2% and 6% at 50 mg/kg and 10 mg/kg dose, respectively and a moderate mean half-life ranging from 3.1 to 4.4 hours.

**Conclusion:**

Even though the oral bioavailability of NP046 is low, its percentage parasite growth inhibition is promising, but in order to improve the oral bioavailability, structure-activity-relationship (SAR) optimization studies are currently being conducted.

**Electronic supplementary material:**

The online version of this article (doi:10.1186/1475-2875-14-8) contains supplementary material, which is available to authorized users.

## Background

Although malaria is completely preventable and treatable [1] it remains a threat to human life and a burden to the global economy due to the emergence of multi-drug resistant malaria parasites.

Artemisinin-based combination therapy (ACT) is recommended as first-line treatment for uncomplicated *Plasmodium falciparum* malaria [2, 3] and has significantly reduced the malaria burden in most endemic countries, but the emergence of artemisinin-resistant malaria parasites in Cambodia, Myanmar, Thailand and Vietnam [1–3] underscores the importance and urgency to push the discovery and development of new effective anti-malarial drugs to its highest level.

Thus to achieve this goal, the Department of Chemistry at the University of the Free State has synthesized a number of novel amino-alkylated chalcones and analogues by means of the Mannich reaction. The compounds were evaluated for their *in vitro* anti-malarial activity against chloroquine-sensitive (D10) and chloroquine-resistant (K1) *P. falciparum* strains, and cytotoxicity against Chinese Hamster Ovarian (CHO) cells by MTT assays [4].

**Figure 1:**
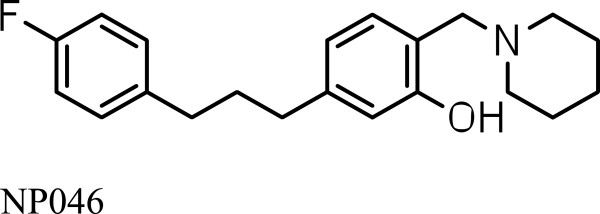
**NP046.**

In this study the *in vivo* efficacy and PK evaluation of the lead compound NP046 (Figure 1) in a mouse model is presented.

## Methods

### Ethics statement

Animal experiments were performed at the animal unit of the PK laboratory of the University of Cape Town, division of clinical pharmacology following the grant of ethical approval from the Animal Research Ethics Committee of the Faculty of Health Sciences of the University of Cape Town (project no. 011/022).

### Reagents and chemicals

NP046 (C_21_H_26_FNO, MW = 327.4) was synthesized and its HPLC purity was determined to be > 99% (for details of the synthesis see Additional file 1). All chemicals and reagents used in this study were of analytical grade or ACS (American Chemical Society) grade. Ammonium formate (97% pure), sodium acetate (purity > 99%), chloroquine phosphate and PEG400 were purchased from Sigma-Aldrich Gmbh (Steinheim, Germany), formic acid (98 – 100%), tert-butylmethyl ether (purity > 99%) and ethanol (GC > 99.9%) were purchased from Merck KGaA (Darmstadt, Germany), acetonitrile and methanol (all of high-purity grade) were purchased from Honeywell, Burdick & Jackson (Muskegon, MI 49442, USA). Water used to prepare solutions was purified by a Millipore Elix 10 reverse osmosis and Milli-Q^®^ (Millipore, USA) Gradient A 10 polishing system.

### Environmental conditions for the animals

Male C57BL/6 mice 12 to 16 weeks old, weighing 20 to 25 g were obtained from the University of Cape Town`s animal unit. They were kept in cages (a maximum of 5 mice per cage) in a temperature-controlled room with a 12 hours day/night light cycle. Ample dried food and water were supplied, and their sanitation was monitored daily. The animals were acclimatized to the test environment for 3 to 4 days before the experiment started.

### *In vivo*efficacy evaluation

*Plasmodium berghei* transfected with green fluorescence protein (strain ANKA), a chloroquine-sensitive strain, was stored in liquid nitrogen. Parasites were thawed and administered intraperitoneally (IP) to infect two donor mice a week before the experiment commenced. Then the parasite-infected red blood cells (RBCs) were collected into heparinized tubes by tail bleeding and the parasitaemia was determined using flow cytometry (≥15%). Finally, each of the test animals received 200 μl of the *P. berghei-*infected RBCs (1×10^7^ per 200 μl PBS) IP to infect them with the parasite. There were three animals per dose group, and each of them received 200 μl of NP046 in water orally and intravenously (volume adjusted relative to the weight of mouse) at their respective dose concentration as shown below:

-  Group A: 50 mg/kg (oral) ≡ 6.25 mg/ml
-  Group B: 10 mg/kg (oral) ≡ 1.25 mg/ml
-  Group C: 5 mg/kg (IV) ≡ 0.625 mg/ml
-  Group D: 1 mg/kg (IV) ≡ 0.125 mg/ml
-  Group E: the negative control group received the drug vehicle alone (placebo)
-  Group F: the positive control group received 10 mg/kg CQ (oral) = 1.25 mg/ml (free base)

The test compound was administered to the animals once a day for four days. The first dose was administered two hours after infection on day one, followed by the second, third and fourth dose at intervals of 24 hours. Blood samples were collected *via* tail bleeding in tubes containing PBS on day 5, and % parasitaemia was determined with FACSCalibur™ using the software CellQuestPro [5].

Finally, the percentage parasite growth inhibition was calculated using the following equation [6]:


### *In vivo*PK evaluation

The *in vivo* PK properties of NP046 were evaluated following oral (50 and 10 mg/kg) and IV (5 mg/kg) administration of the compound in water to male C57BL/6 mice (n = 5). Blood samples were collected *via* tail bleeding in heparinized tubes at predetermined sampling times on ice, and were stored at -80°C until analysis. Despite the critical problem of obtaining numerous blood samples from a mouse without compromising the health/safety of the animal, blood samples at all the sampling times, i.e. 0.17, 0.5, 1, 3, 5 and 7 hours for the IV dose groups, and 0.5, 1, 3, 5 and 7 hours for the oral dose groups were collected from each mouse. The extraction volume during sample analysis was targeted at the lowest possible volume (20 μl) thereby minimizing the total volume of blood harvested from the animal.

Blood samples collected from mice following the administration of NP046 together with the calibration standards (STDs) and quality controls (QCs) of NP046 were analyzed using a validated LC-MS/MS assay (for details of the LC-MS/MS assay see Additional file 2). Concentration *vs* time profiles were constructed and using Summit PK software the data were processed to obtain the PK properties of NP046.

## Results and discussion

### *In vivo*efficacy evaluation

**Figure 2:**
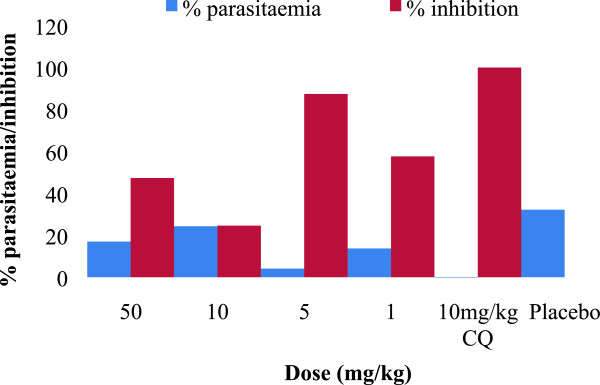
**% parasite growth inhibition chart for NP046 in** ***P. berghei-*** **infected mice (n = 5) on day five post-infection.**

The mean percentage parasitaemia (± S.E.M) and percentage of parasite growth inhibition data of NP046 in *P. berghei*-infected mice assessed on day five post-infection is presented in Table 1, and the summary chart of the percentage of parasite growth inhibition is presented in Figure 2. The 50 and 10 mg/kg NP046 oral dose groups resulted in percentage of parasite growth inhibition of 47.3% and 24.6%, respectively, while the 5 and 1 mg/kg NP046 IV dose groups resulted in 87.4% and 57.6% growth inhibition, respectively, on day five post-infection relative to the negative control (placebo) group. The IV dose group showed a higher percentage of parasite growth inhibition relative to the oral dose group, which correlates well with the poor oral absorption of NP046 as presented in the next section.

**Table 1:** **Mean percentage of parasitaemia (± S.E.M) and percentage of parasite growth inhibition data of NP046 in** ***P. berghei-*** **infected male C57BL/6 mice, assessed on day five post-infection**

Treatment	% parasitaemia	% growth inhibition
**50 mg/kg NP046 oral**	16.7 ± 3.34	47.3
**10 mg/kg NP046 oral**	24.3 ± 0.80	24.6
**5 mg/kg NP046 IV**	4.05 ± 0.12	87.4
**1 mg/kg NP046 IV**	10.2 ± 1.75	57.6
**Positive control**	0.01 ± 0.0	100
**Negative control**	32.2 ± 0.50	0

Compounds that reduce parasitaemia by 30% or more are considered active and are further evaluated in secondary screens [7]. Thus, NP046 which exhibited a 47.3% reduction in parasitaemia at a 50 mg/kg oral dose relative to CQ can be considered as an active compound and proceed for further secondary screens.

### *In vivo*PK evaluation

**Figure 3:**
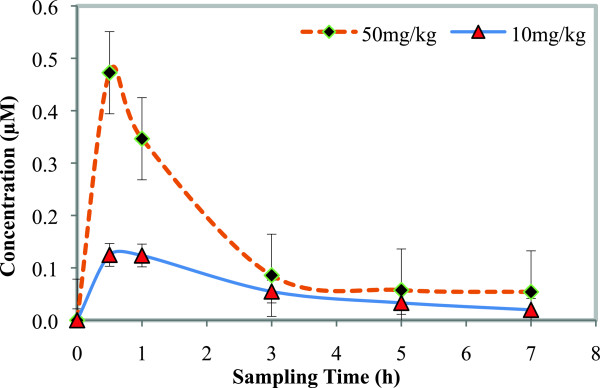
**Mean blood concentration** ***vs*** **time profiles of NP046 following oral administration of 50 and 10 mg/kg NP046, respectively, to male C57BL/6 mice (n = 5).**

**Figure 4:**
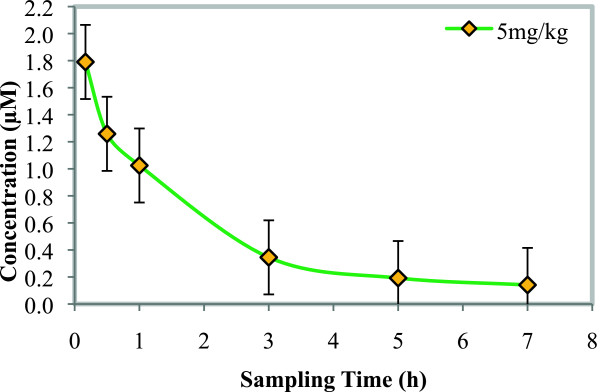
**Mean blood concentration** ***vs*** **time profiles of NP046 following IV administration of 5 mg/kg NP046 to male C57BL/6 mice (n = 5).**

Blood samples collected from mice after the administration of NP046, together with the calibration standards (STDs) and quality controls (QCs), were analysed using a fully validated LC-MS/MS assay. NP046 concentrations were detectable for up to seven hours after oral and IV dosing. Concentration *vs* time profiles (Figures 3 and 4) were constructed and the data processed with Summit PK software (non-compartmental analysis) to obtain the PK parameters as presented in Table 2. The LC-MS/MS method performed very well; NP046 concentrations in the mice were detectable for up to seven hours after both 50 and 10 mg/kg oral dosing, with mean concentrations of 0.05 and 0.02 μM, respectively. In contrast to oral dosing, the 5 mg/kg IV dose resulted in a mean blood NP046 concentration at seven hours after dosing of 0.14 μM.

**Table 2:** **PK parameters of NP046**

PK parameter	Oral		IV
**Nominal Dose (mg/kg)**	50	10	5
**Apparent t**_**1/2**_ **(h)**	4.4 ± 0.60	3.1 ± 0.46	3.2 ± 0.35
**Blood CL**_**total**_ **(mL/min/kg)**	—	—	62.2 ± 3.0
**Vd (L/Kg)**	—	—	16.9 ± 1.3
**C**_**max**_ **(μM)**	0.47 ± 0.10	0.13 ± 0.06	—
**T**_**max**_ **(min)**	30	48	—
**AUC**_**0 - ∞**_ **(min. μmol/L)**	80 ± 8.8	30 ± 14.7	248 ± 12.6
**Bioavailability (%)**	3.2 ± 0.47	6.0 ± 2.9	N/A

Results are mean of n = 5 ± S.E.M.

The oral dose regimens of NP046 showed dose-dependent PK relationships, i.e. increasing the oral dose from 10 mg/kg to 50 mg/kg increased the AUC from 30 to 80 min.μmol/L. The mean elimination half-life of NP046 following oral administration was 4.4 hours and 3.1 hours for the 50 mg/kg and 10 mg/kg doses, respectively, while for the IV route it was 3.2 hours, indicating a moderate elimination half-life. The compound has a very high volume of distribution (16.9 L/kg), indicating high distribution of the drug in tissues. The estimated oral bioavailability of NP046 at 50 mg/kg and 10 mg/kg were very low at 3.2% and 6.0%, respectively.

The oral bioavailability of NP046 was low. Low oral bioavailability could be the result of the following reasons [8]:

-  first-pass metabolism at the intestinal wall or portal circulation to the liver, both common sites of first-pass metabolism;
-  poor water solubility of drug which results in slow absorption, and thus, low bioavailability;
-  membrane permeability; and
-  chemical interactions, such as hydrolysis by the gastric acid or enzyme, complex formation, conjugation in the intestinal wall, etc., which affect absorption could also affect bioavailability.

Therefore, investigating some of the reasons for low bioavailability in the early stage of the drug development process, such as plasma-protein binding, microsomal stability, solubility, etc., is helpful in enhancing the drug development process. Thus, in this study the following investigations on some of the probable causes for low bioavailability were performed:

-  plasma protein binding assessment;
-  kinetic solubility;
-  microsomal stability;
-  lipophilicity; and
-  permeability.

### Plasma protein binding assessment

PPB (plasma protein binding), the extent to which a drug binds to protein in plasma, can affect the PK-PD properties of a drug. Thus, determining the PPB property of a drug in the early stage of drug development process is crucial and a prerequisite for lead prioritization [9]. Initially the PPB assay was performed using an ultrafiltration method [see Additional file 3], which depends on centrifugal force to move the unbound analyte of interest through a size-selective membrane. Ultrafiltration is recommended for the determination of PPB property if the non-specific binding (NSB) of the test drug to the micro-partition device is less than 5%. If NSB > 5%, an alternative technique such as equilibrium dialysis or ultracentrifugation should be used [9].

In this study, since the NSB > 5%, ultracentrifugation was used to assess the PPB property of NP046 [for the methodology see Additional file 3]. NP046 showed a moderate PPB property, i.e. 28% at 1000 ng/ml and 20.3% at 10 ng/ml.

### Kinetic solubility

Kinetic solubility is the measurement of the solubility of a pre-dissolved compound (typically in DMSO, a strong organic solvent) in an aqueous buffer [10]. Solubility, one of the most important properties in a drug discovery process, is a determinant factor in intestinal absorption and oral bioavailability. In other words, for a drug to be absorbed and became available in the systemic circulation and to act on the target cell, irrespective of its formulation or route of administration, it should go into solution, i.e. it should be able to dissolve and diffuse through the intestinal membrane [10]. The kinetic solubility of NP046 was determined using an HPLC-UV method at pH 2.0 and 7.4 [for the methodology see Additional file 4]. The concentration of the test compound (x) in PBS samples was calculated from the peak area (y) in its UV chromatogram by extrapolation from the three-point calibration line, using the equation *y* = *ax* + *b*; where a = slope and b = intercept. The results are presented in Table 3.

**Table 3:** **Kinetic solubility of NP046 and controls**

Compounds	Solubility (μM)	
	pH 2.0	pH 7.4
**Reserpine**	192.8	-4.2
**Hydrocortisone**	190.6	185.9
**NP046**	185.3	-2.5

The pH of the stomach is approximately 1.4 to 2.0, and that of the intestine where drug absorption takes place is approximately pH 6.8 to 8.0 [11]. NP046 is highly soluble at pH 2.0 indicating that it can dissolve in the stomach and pass into the intestine (pH 6.8 – 8) where it starts to precipitate as its solubility at pH 7.4 is very low. Thus its ability to diffuse through the intestinal membrane and get absorbed is limited resulting in a very low oral bioavailability as shown in the PK evaluation experiment.

### Metabolic stability

Metabolism is an enzymatic process in which a drug is chemically modified into polar metabolites that can be more readily excreted from the body. Metabolism occurs mainly in the liver, and the intestine [10]. The metabolic stability of NP046 was determined in human and mouse liver microsomes using a multiple-time-point method at 0, 5, 10, 30, and 60 minutes [for the methodology see Additional file 5]. The *in vitro* intrinsic clearance values of NP046 in human and mouse liver microsomes were 118.7 μl/min/mg and 656.8 μl/min/mg, respectively, which are considered high and require a metabolism SAR optimization of NP046 to improve its metabolic stability.

### Permeability

Permeability is the speed/velocity and extent to which a drug passes through a biological membrane obstacle in a living system, such as the gastrointestinal (GI) epithelial cells, the blood capillary wall, the target cell membrane [10]. The Caco-2 cell line (a continuous line of heterogeneous human epithelial colorectal adenocarcinoma cells), the most widely used and recognized as a model of the intestinal barrier [11] was used to determine the *in vitro* permeability of NP046 [for the methodology see Additional file 6].

The transport of each drug from the apical side to the basolateral side was done using a transport buffer that consisted of Hank’s Balanced Salt Solution (HBSS) + 10 mM HEPES + 0.35 g/ml NaHCO_3_, pH 7.4 (1:100 1 M HEPES in HBSS). The drug transport assay was performed by determining the concentrations of the test compound from the basolateral chamber, using an LC-MS/MS assay. But no quantifiable amount of the test compound was found in the solution obtained from the basolateral chamber. Therefore, the experiment was repeated and the same result was obtained. This could be due to the following reasons:

-  the compound was bound to the membrane of the device (6.5 mm Transwell with 0.4 μm pore polycarbonate membrane insert) as it happened during plasma protein binding assessment using the ultrafiltration method, i.e., high NSB to the membrane of the device; or
-  a solubility problem, in that the test compound showed very low solubility at pH 7.4. Therefore, as the pH of the transporter buffer was 7.4, the test compound might have formed a precipitate resulting in poor permeability.

### Lipophilicity

Lipophilicity is a compound’s tendency to partition into a non-polar lipid matrix versus an aqueous matrix [10]. It is an important property of a drug which has a significant influence on the drug ADME/tox properties as well as its biological activity. In this paper, the distribution coefficients (Log D_pHx_) of NP046 and controls (ouabain, hydrocortisone and verapamil) were determined using a scaled-down shake-flask method that had been adapted for a 96-well plate in order to enable a high throughput.

Stock solutions of NP046 and controls in DMSO (10 mM) were prepared and an aliquot of each phase (octanol/PBS) was analysed by an HPLC-Diode array detector (DAD) method. Chromatographic separation was achieved on a Phenomenex^®^ Kinetex C18 (50 × 2.1 mm i.d. 2.6 μm) column with a gradient elution, using mobile phase (A) 5 mM ammonium format-acetonitrile (95:5, v/v) and (B) 5 mM ammonium format-acetonitrile (5:95, v/v). The Log D values were calculated using the analyte peak areas obtained from each phase (Octanol/PBS) according to the following equation [12]:


The Log D_7.4_ for NP046 was found to be 2.6, which is in the ideal range (Log D_7.4_ 1 to 3), and is expected to have good intestinal absorption but minimum metabolism. However, its oral bioavailability was low which could have resulted from intestinal metabolism leading to first-pass extraction. The small intestine, which is estimated to have about 1% CYP3A of the average hepatic content, has the ability to metabolise drugs resulting in first-pass extraction more than the hepatic metabolism for some drugs leading to limited oral bioavailability [13].

## Conclusion

Even though the oral bioavailability of NP046 is very low, its parasite growth inhibition was moderate at a 50 mg/kg oral dose and high at a 5 mg/kg IV dose, i.e. 47.3% and 87.4%, respectively. The lower parasite growth inhibition with oral dosing relative to the IV dosing correlates well with poor oral bioavailability. A SAR optimization process has been triggered by these results and a next generation of analogues has been synthesized by the Department of Chemistry of the University of the Free State, and is currently being evaluated for their bioavailability and efficacy properties.

## Electronic supplementary material

Additional file 1: **The synthesis of nitrogen containing chalcones and analogues.** The data provided describes the method used to synthesize the nitrogen containing chalcones and analogues, and the resulting NMR and IR data. (DOCX 24 KB)

Additional file 2: **LC-MS/MS assay for the analysis of NP046.** The data provided describes the detection and chromatography, sample preparation and some validation results from the LC-MS/MS assay used to analyse NP046 PK-samples. (DOCX 136 KB)

Additional file 3: **Plasma protein binding (PPB) assay procedure.** The data provided describes the procedure used for the determination of nonspecific binding of the test drug to the micro-partitioning device followed by the PPB-assay using ultrafiltration and ultracentrifugation methods. (DOCX 21 KB)

Additional file 4: **Summary of the assay method for the determination of kinetic solubility.** The data provided describes the assay procedure used for the determination of the kinetic solubility of the test drug. (DOCX 21 KB)

Additional file 5: **Summary of the assay method for the determination of microsomal stability.** The data provided describes the assay procedure used for the determination of the microsomal stability of the test drug in human and mouse microsomes. (DOCX 24 KB)

Additional file 6: **Caco-2 permeability assay protocol.** The data provided describes the assay procedure used for the determination of the permeability of the test drug through a biological membrane. (DOCX 22 KB)

## Abbreviations

PPB: Plasma Protein Binding
NSB: Non-Specific Binding
GI: Gastrointestinal
ACS: American chemical society
AUC: Area under the curve
CHO: Chinese hamster ovarian
C_max_: Maximum concentration
CQ: Chloroquine
CV: Coefficient of variation
EMA: European Medicines Agency
FDA: Food and Drug Administration
HPLC: High performance liquid chromatography
IS-MF: Internal standard normalized matrix factor
IV: Intravenous
LC-MS/MS: Liquid chromatography tandem mass spectrometer
LLE: Liquid-liquid extraction
LLOQ: Lower limit of quantification
MRM: Multiple reaction monitoring
MTT: (3-(4, 5-Dimethylthiazol-2-yl)-2, 5-diphenyl tetrazolium bromide
Nom: Nominal
PK: Pharmacokinetic
STD: Standard
QC: Quality control
S/N: Signal-to-Noise ratio
ULOQ: Upper limit of quantification
PBS: Phosphate buffer saline
SAR: Structure-Activity-Relationship
ACT: Artemisinin-based combination therapy
FACS: Fluorescence activated cell sorters, S.E.M, Standard Error of the Mean.
