# Supplementary material for: Efficacy and pharmacokinetic evaluation of a novel anti-malarial compound (NP046) in a mouse model
Source: Malar J. 2015 Jan 6;14:8. doi: 10.1186/1475-2875-14-8 (PMC4326489; doi:10.1186/1475-2875-14-8)
Supplement: Supplementary file 1 — Additional file 1: The synthesis of nitrogen containing chalcones and analogues. The data provided describes the method used to synthesize the nitrogen containing chalcones and analogues, and the resulting NMR and IR data. (DOCX 24 KB) [file 12936_2014_3683_MOESM1_ESM.docx]

**The synthesis of nitrogen containing chalcones and analogues**

1. **Synthesis of (*E*)-1-(4-fluorophenyl)-3-(3-hydroxyphenyl) prop-2-en-1-one**

4-Fluoroacetophenone (1.000 g; 7.2 mmol) and 3-hydroxybenzaldehyde (0.883 g; 7.2 mmol) were dissolved in EtOH (50 mL) with constant stirring at room temperature. A 10% NaOH solution (50 mL) was added after 10 minutes, which turned the reaction mixture bright yellow. The reaction was left to stir overnight. The reaction mixture was poured over ice and 1 N HCl (25ml) solution and extracted with EtOAc, dried over Na_2_SO_4_ and evaporated under reduced pressure. The chalcone was isolated by column chromatography (T:A 5:5, 3 cm x 30 cm).

The fraction R_f_ 0.52 yielded (*E*)-1-(4-fluorophenyl)-3-(3-hydroxyphenyl)prop-2-en-1-one (**1**) as a yellow solid^^[[1]](#footnote-1)^^ (1.56 g, 90%). ^1^H NMR δ (600 MHz, CDCl_3_, Me_4_Si) 8.04 (2H, dd, ^3^J_H-H_ = 8.8 Hz; ^4^J_H-F_ = 5.4 Hz, H-2', H-6'), 7.75 (1H, d, J = 15.6 Hz, H-3), 7.46 (1H, d, J = 15.6 Hz, H-2), 7.28 (1H, t, J = 7.8 Hz, H-5''), 7.20 (1H, d, J = 7.7 Hz, H-4''), 7.16 (2H, t, ^3^J_H-H_ = 8.8 Hz; ^4^J_H-F_ = 8.8 Hz, H-3', H-5'), 7.14 – 7.12 (1H, m, H-2''), 6.92 – 6.89 (1H, m, H-6''). ^13^C NMR δ (150 MHz, CDCl_3_, Me_4_Si) 189.1 (C-1), 165.7 (1C, d, ^1^J_CF_ = 255.1 Hz, C-4'), 156.2 (C-3''), 144.9 (C-3), 136.4 (C-1''), 134.4 (1C, d, ^4^J_CF_ = 2.5 Hz, C-1'), 131.2 (2C, d, ^3^J_CF_ = 9.2 Hz, C-2', C-6'), 130.3 (C-5''), 121.9 (C-2), 121.2 (C-6''), 117.9 (C-2''), 115.9 (2C, d, ^2^J_CF_ = 21.8 Hz, C-3', C-5'), 115.0 (C-4'').

IR (neat): ν_max_ = 1577.77, 1507.52, 857.59, 811.33, 572.79 cm^-1^

1. **Synthesis of 3-(3-(4-fluorophenyl)propyl)phenol**

(*E*)-1-(4-fluorophenyl)-3-(3-hydroxyphenyl)prop-2-en-1-one (**1**) (0.500 g, 2.07 mmol) was dissolved in a 1:3 (v/v) solution of EtOAc:H_2_O. 10% aq. HCl (10 mL) with Pd(OH)_2_/C (0.060 g) was added and the system flushed with hydrogen. The reaction mixture was left to stir at room temperature for 48–72 hrs under H_2_ at atomspheric pressure. After completion of the reaction (TLC) the reaction mixture was filtered through silica gel, the filtrate extracted with EtOAc (2 x 50 mL) and washed with water (1 x 30 mL) and brine (1 x 20 mL). The organic layer was dried over anhydrous MgSO_4_, and the solvent evaporated under reduced pressure. The resulting crude mixture was chromatographed on a silica gel column (7:3 Hexane:EtOAc).

The fraction R_f_ 0.50 yielded 3-(3-(4-fluorophenyl)propyl)phenol (**2**) as a yellow oil (0.410 g, 86%). ^1^H NMR δ (600 MHz, CDCl_3_, Me_4_Si) 7.15 – 7.09 (1H, m, H-5''), 7.09 (2H, dd, ^3^J_H-H_ = 8.6 Hz; ^4^J_H-F_ = 5.5 Hz, H-2', H-6'), 6.94 (2H, t, ^3^J_H-H_ = 8.6 Hz; ^4^J_H-F_ = 8.6 Hz, H-3', H-5'), 6.73 (1H, d, J = 7.6 Hz, H-4''), 6.68 – 6.64 (2H, m, H-2'', H-6''), 2.59 – 2.56 (2H, m, H-1), 2.56 – 2.53 (2H, m, H-3), 1.91 – 1.84 (2H, m, H-2). ^13^C NMR δ (150 MHz, CDCl_3_, Me_4_Si) 161.3 (1C, d, ^1^J_C-F_ = 242.8 Hz, C-4'), 155.7 (C-3''), 144.1 (C-1''), 137.9 (1C, d, ^4^J_C-F_ = 3.2 Hz, C-1'), 129.8 (2C, d, ^3^J_C-F_ = 7.8 Hz, C-2', C-6'), 129.6 (C-5''), 120.9 (C-6''), 115.5 (C-2''), 115.1 (2C, d, ^2^J_C-F_ = 20.9 Hz, C-3', C-5'), 112.9 (C-4''), 35.2 (C-1), 34.6 (C-3), 32.9 (C-2). ^19^F NMR δ (282.4 MHz, CDCl_3_, C_6_F_6_) -117.9 (s, F).

IR (neat): ν_max_ = 2938.38, 1508.14, 1218.37, 1154.84, 834.11, 696.82 cm^-1^

1. **Synthesis of 5-(3-(4-fluorophenyl)propyl)-2-(piperidin-1-ylmethyl)phenol**

3-(3-(4-fluorophenyl)propyl)phenol (**2**) (0.200 g; 0.87 mmol), paraformaldehyde (0.052 g; 1.73 mmol), and piperidine (0.18 mL; 1.84 mmol) were dissolved in EtOH (2 mL) and conc. HCl (5 drops). The reaction mixture was refluxed for 9 hours until TLC showed the disappearance of the starting material. The reaction mixture was quenched with solid NaHCO_3_, extracted with EtOAc (2 x 50 mL) and washed with water (2 x 50 mL). The organic layer was dried over Na_2_SO_4_ and the solvent evaporated under reduced pressure. The crude reaction mixture was chromatographed (TLC, T:A 7:3).

The fraction R_f_ 0.50 yielded 5-(3-(4-fluorophenyl)propyl)-2-(piperidin-1-ylmethyl)phenol (**3**) as a yellow oil (0.269 g, 95%). ^1^H NMR δ (600 MHz, CDCl_3_, Me_4_Si) 7.05 (2H, dd, ^3^J_H-H_ = 8.6 Hz; ^4^J_H-F_ = 5.5 Hz, H-2', H-6'), 6.87 (2H, t, ^3^J_H-H_ = 8.6 Hz; ^4^J_H-F_ = 8.6 Hz, H-3', H-5'), 6.78 (1H, d, J = 7.6 Hz, H-5''), 6.57 (1H, d, J = 1.3 Hz, H-2''), 6.50 (1H, dd, J = 7.6, 1.3 Hz, H-6''), 3.55 (2H, s, C**H_2_**), 2.55 – 2.51 (2H, m, H-3), 2.50 – 2.46 (2H, m, H-2), 2.41 – 2.04 (4H, H-2''', H-6'''), 1.87 – 1.78 (2H, m, H-2), 1.64 – 1.32 (6H, H-3''', H-4''', H-5'''). ^13^C APT NMR δ (150 MHz, CDCl_3_, Me_4_Si) 161.2 (1C, d, ^1^J_C-F_ = 243.1 Hz, C-4'), 158.0 (C-3''), 142.8 (C-1''), 138.0 (1C, d, ^4^J_C-F_ = 3.2 Hz, C-1'), 129.7 (2C, d, ^3^J_C-F_ = 7.7 Hz, C-2', C-6'), 128.3 (C-2''), 119.1 (C-4''), 119.0 (C-6''), 116.0 (C-2''), 115.0 (2C, d, ^2^J_C-F_ = 20.9 Hz, C-3', C-5'), 61.9 (**C**H_2_), 53.9 (C-2''', C-6'''), 35.1 (C-3), 34.6 (C-1), 32.9 (C-2), 25.9 (C-3''', C-5'''), 24.0 (C-4'''). ^19^F NMR δ (282.4 MHz, CDCl_3_, C_6_F_6_) -118.1 (s, F).

IR (neat): ν_max_ = 2934.84, 1508.34, 1218.86, 819.83 cm^-1^

Found (TOF MS ES) [M+H]^+^ 328.2073, (C_21_H_26_FNO + H^+^) requires *m/z* 328.2077.

HPLC purity 96.9%, t_R_ = 1.62 min.

1. Van Der Westhuizen, J. H.; Eljaleel, A. E. M.; Bonnet, S. L.; Wilhelm-Mouton, A. *PCT Int. Appl*. 2011, WO 2011151789 A2 20111208. [↑](#footnote-ref-1)
