# Supplementary material for: Efficacy and pharmacokinetic evaluation of a novel anti-malarial compound (NP046) in a mouse model
Source: Malar J. 2015 Jan 6;14:8. doi: 10.1186/1475-2875-14-8 (PMC4326489; doi:10.1186/1475-2875-14-8)
Supplement: Supplementary file 2 — Additional file 2: LC-MS/MS assay for the analysis of NP046. The data provided describes the detection and chromatography, sample preparation and some validation results from the LC-MS/MS assay used to analyse NP046 PK-samples. (DOCX 136 KB) [file 12936_2014_3683_MOESM2_ESM.docx]

## LC-MS/MS assay for the analysis of NP046

The method was validated in accordance to the FDA guidance for industry, 2001 [1] and EMA guideline on bioanalytical method development, 2012 [2].

### Matrix

Owing to the scarcity of mouse blood, the assay was developed and validated using human whole blood and then cross validated with mice whole blood to ensure that the same results were obtained in the same matrix of both species.

### Detection and chromatography

An AB SCIEX API 3200 triple quadrupole mass spectrometer, equipped with ESI source operated at 600^o^C was used to analyse NP046. ESI in the positive ion mode was used for ion production. Transition of the protonated precursor ion m/z 328, and m/z 450 to the product ion m/z 243 and m/z 324 for NP046 and the internal standard, respectively were monitored at a unit resolution in the multiple reaction monitoring (MRM) mode (see the infusion product ion mass spectrum of NP046 and the internal standard in Figures 1 and 2, respectively).


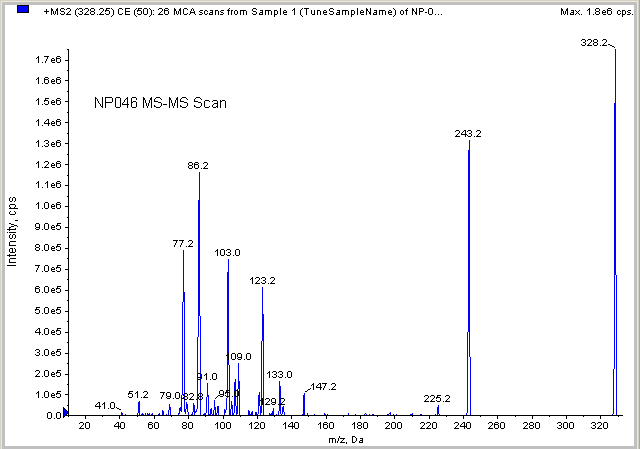


**Figure 1** Infusion product ion mass spectrum of NP046


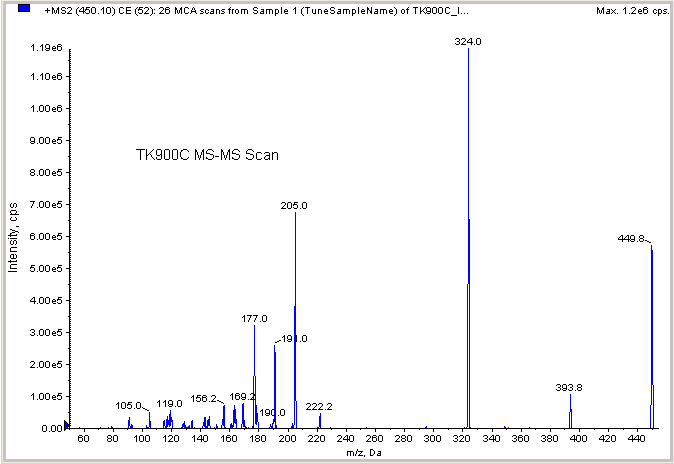


**Figure 2:** Infusion product ion mass spectrum of TK900C

The instrument was interfaced to a work station running Analyst^TM^ version 1.5.2 software and all data were captured and stored electronically on the work station’s hard disc drive.

HPLC analysis was performed with an Agilent 1200 infinity series quaternary pump delivering the mobile phase at a flow rate of 200 µl/min combined with an Agilent 1200 infinity series autosampler, degasser, and column compartment. The autosampler was equipped with a 96-well plate holder and used to inject 5µl of each sample onto the HPLC column. An Agilent cooling device was set at ~ 5^o^C to keep the samples cool. Chromatography was performed on a Phenomenex^®^ Kinetex C18 (100 x 2.0 mm id, 2.6 µm) analytical column fitted with a Phenomenex^®^ Security Guard^TM^ System that contained a C18 (4 x 3 mm) precolumn. The column was kept at 30^o^C with an Agilent 1200 infinity series column compartment. The ion source and mass spectrometer settings are given below in Tables 1 and 2.

**Table 1:** Ionization source setting for NP046

| **Electro Spray Ionisation Settings** | **Value** |
| --- | --- |
| Nebulizer gas (Gas 1) (arbitrary unit) | 30 |
| Turbo gas (Gas 2) (arbitrary unit) | 30 |
| CUR (curtain gas) (arbitrary unit) | 20 |
| CAD (collision gas) (arbitrary unit) | 5 |
| TEM (source temperature) (°C) | 600 |
| IS ( Ion Spray Voltage) (V) | 2000 |

**Table 2:** MS/MS detector setting for NP046

| **MS/MS Settings** | **NP046** | **ISTD (TK900C)** |
| --- | --- | --- |
| Monoisotopic mass | 327.1998 | 449.2095 |
| Protonated molecular mass (*m/z*) | 328 | 450 |
| Product ion molecular mass (*m/z*) | 243 | 324 |
| Dwell time (ms) | 150 | 150 |
| DP (declustering potential) (V) | 50 | 50 |
| EP (entrance potential) (V) | 7.5 | 7.5 |
| CE (collision energy) (eV) | 27 | 31 |
| CXP (collision cell exit potential) (V) | 6 | 6 |
| Scan type | MRM | MRM |
| Polarity | Positive | Positive |
| Pause time | 5ms | 5ms |

### Sample preparation

Blood samples were completely thawed unassisted at room temperature and briefly vortexed, followed by centrifugation at 1300 g for three minutes. The analyte and ISTD were isolated using LLE as described below.

**Extraction procedure:**

1. Aliquot 50 µl of a 20 mM ammonium formate buffer, pH 7.0, into a 2 ml microfuge tube.
2. Add 20 µl blood sample into the buffer.
3. Add ISTD solution (100 µl, 50 ng/ml of TK900C in water).
4. Add *tert*-butyl methyl ether (1 ml).
5. Vortex for 2 minutes and centrifuge at 2000 g for five minutes.
6. Freeze the aqueous phase in an alcohol freezing bath (~ -20^o^C).
7. Decant the organic phase into a clean 5 ml polypropylene tube.
8. Evaporate to dryness under a gentle stream of nitrogen at ~ 40^o^C.
9. Add 0.1% formic acid (400 µl).
10. Vortex for 40 seconds.
11. Transfer extracts to a 96-well plate and inject 5 µl onto the HPLC column.

### Assay specificity

Blank human blood samples obtained from ten different sources were tested for any visible interference. A representative chromatogram of a blank extract, as shown in Figure 3, indicates that there was no interference, i.e. no endogenous peaks at or near the retention time of the analyte or the internal standard.


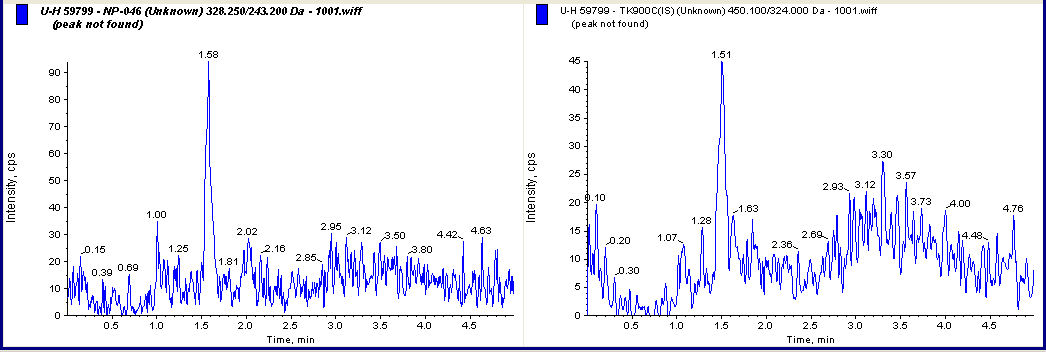


**Figure 3:** Representative chromatogram of a blank human whole blood extract from the NP046 selectivity assessment

### Linearity and LLOQ

The method was validated by analysing the blood QCs at nine concentration levels in replicates of six to determine the accuracy and precision of the method. The QC values were calculated from a standard regression curve with ten concentration levels (2.00 to 1000 ng/ml). A calibration graph was constructed using a WAGNER (LOG-LOG QUADRATIC) curve fit with weighting factor 1 based on peak area ratio as the response type (ln(y) = a(ln(x))^2^ + b(ln(x)) + c) and r^2^ of 0.9991. The cumulative results of three representative standard curves for NP046 are presented in Table 3. A representative calibration curve, chromatogram at LLOQ (STD B), and oral and IV samples collected 30 min after dosing are presented in figures 4, 5, 6, and 7, respectively.


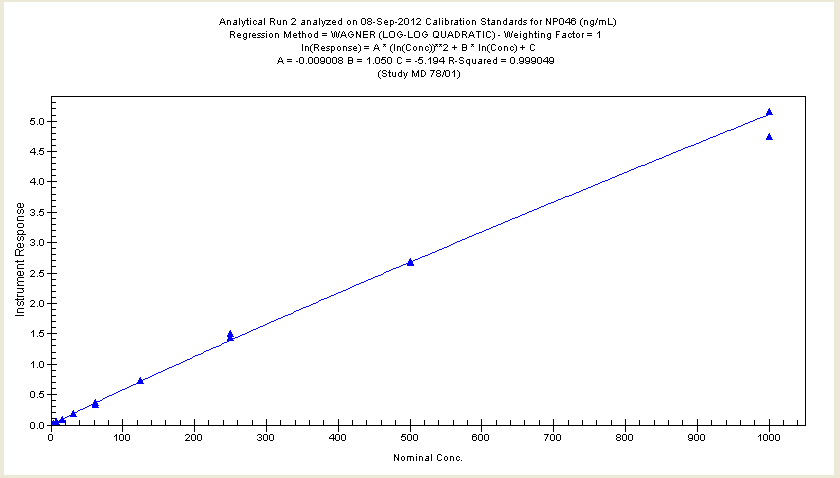


**Figure 4**: Calibration curve for validation run-1


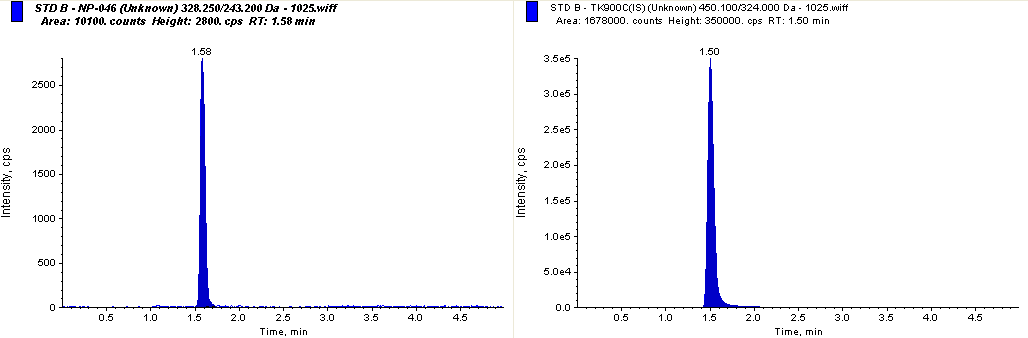


**Figure 5**: Representative chromatogram at LLOQ (STD B)


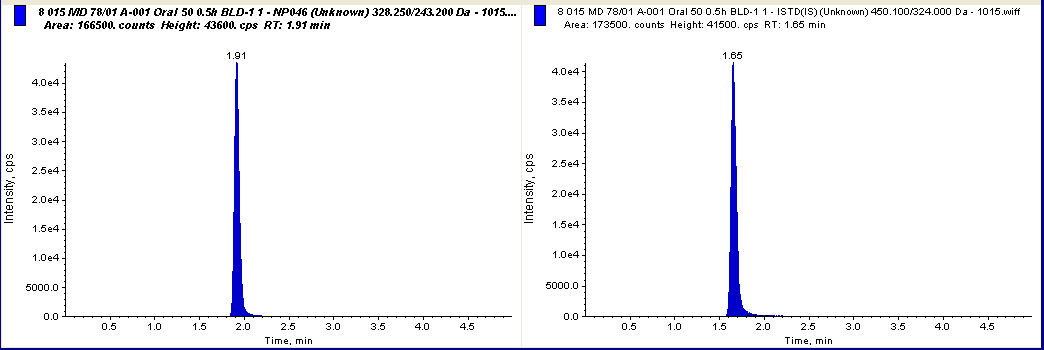


**Figure 6**: Representative chromatogram of mice blood sample 30 min after oral dosing


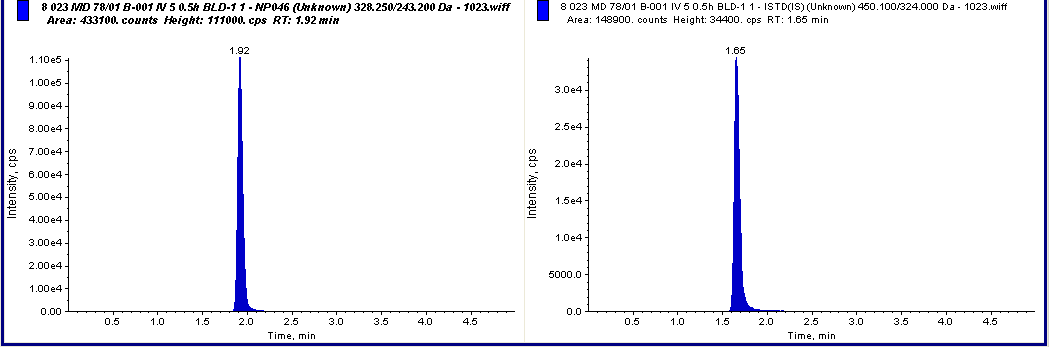


**Figure 7**: Representative chromatogram of mice blood sample 30 min after IV dosing

**Table 3** Cumulative statistics of NP046 calibration standards and quality control samples

| Parameters | Calibration standards and nominal concentrations (ng/ml) | | | | | | | | | | |  |
| --- | --- | --- | --- | --- | --- | --- | --- | --- | --- | --- | --- | --- |
|  | STD B | STD C | STD D | STD E | STD F | STD G | STD H | STD I | STD J | STD K | N/A |  |
|  | 2.000 | 3.999 | 7.998 | 16.00 | 31.99 | 62.50 | 125.0 | 250.0 | 500.0 | 1000 | N/A |  |
| Mean | 2.018 | 4.057 | 8.032 | 15.59 | 31.28 | 63.18 | 129.0 | 254.0 | 505.8 | 974.0 | N/A |  |
| %Nom | 100.9 | 101.5 | 100.4 | 97.5 | 97.8 | 101.1 | 103.2 | 101.6 | 101.2 | 97.4 | N/A |  |
| % CV | 9.8 | 9.1 | 5.7 | 6.4 | 8.7 | 5.1 | 3.6 | 4.4 | 7.9 | 3.6 | N/A |  |
| % Bias | 0.9 | 1.5 | 0.4 | -2.5 | -2.2 | 1.1 | 3.2 | 1.6 | 1.2 | -2.6 | N/A |  |
| N | 6 | 6 | 6 | 6 | 6 | 6 | 6 | 6 | 6 | 6 | N/A |  |
| Parameters | Quality control samples and nominal concentration (ng/ml) | | | | | | | | | | |  |
|  | QC A | QC B | QC C | QC D | QC E | QC F | QC G | QC H | | QC I | QC J DIL |  |
|  | 2.000 | 4.999 | 10.01 | 20.00 | 40.00 | 100.0 | 200.0 | 400.0 | | 799.9 | 1600 |  |
| Mean | 1.973 | 5.037 | 9.065 | 19.72 | 38.98 | 96.6 | 199.4 | 383.7 | | 739.6 | 1492 |  |
| %Nom | 98.7 | 100.8 | 90.6 | 98.6 | 97.5 | 96.6 | 99.7 | 95.9 | | 92.5 | 93.2 |  |
| %CV | 9.5 | 8.2 | 6.7 | 8.4 | 10.3 | 5.8 | 6.8 | 6.5 | | 6.5 | 7.0 |  |
| %Bias | -1.3 | 0.8 | -9.4 | -1.4 | -2.5 | -3.4 | -0.3 | -4.1 | | -7.5 | -6.8 |  |
| N | 18 | 18 | 18 | 18 | 18 | 18 | 18 | 18 | | 18 | 6 |  |

QCJ DIL was used to establish the dilution linearity of the method

Precision and accuracy

The within- and between-batch accuracy (%Nom) and precision (%CV) of the assay procedure were assessed by calculating the accuracy and precision statistics of the 9 levels of quality control standards (n = 6 per batch) over all three validation runs, as presented in Table 3. The deviation is within ± 15% of the nominal value at all the concentration levels. This indicates an acceptable accuracy and precision.

Extraction efficiency

The extraction recovery determined for NP046 was consistent and repeatable. The results are presented in Table 4.

**Table 4** Absolute recovery, using response factor

| Sample | Analyte conc. (ng/ml) | Mean of peak areas | | Absolute recovery (%) | CV (%) |
| --- | --- | --- | --- | --- | --- |
|  |  | After extraction | Theoretical values |  |  |
| High conc. | 799.9 | 1046183 | 1617021 | 64.7 | 8.7 |
| Medium conc. | 100.0 | 136817 | 219419 | 62.4 | 12.2 |
| Low conc. | 4.999 | 6854 | 11170 | 61.4 | 10.4 |
| Mean | | | | 62.8 | 10.4 |
| ISTD | 50.00 | 142983 | 210410 | 68.0 | 14.4 |

N.B.: The concentration of the ISTD was same at high, medium and low concentration levels.

Stability assessment

A summary of the stability assessment is presented in Table 5. NP046 was stable for 100 days in stock solutions of methanol and acetonitrile when stored at room temperature and -20^o^C. It was stable in whole blood on bench for 3.3 hours while waiting for extraction, and was stable after three freeze-thaw cycles too. Long term matrix stability showed that NP046 was stable in whole blood at -80^o^C for 92 days.

**Table 5** Stability assessment

| Stability | Analyte code | | Mean analyte peak area (n = 6) | | | |
| --- | --- | --- | --- | --- | --- | --- |
|  |  |  | Room temperature | ~ 5°C | ~ -20°C | Fresh ( reference) |
| Analyte stock solution stability | In methanol | Peak area | 5923000 | 5548000 | 5962000 | 6323000 |
|  |  | % Reference | 93.7 | 87.8 | 94.3 | N/A |
|  |  | % CV | 2.5 | 1.5 | 1.6 | 1.1 |
|  | In acetonitrile | Peak area | 7118333 | 5792833 | 6967500 | 6974167 |
|  |  | % Reference | 102.1 | 83.1 | 99.9 | N/A |
|  |  | % CV | 1.4 | 2.6 | 0.9 | 1.5 |
| Stability | | | NP046 Nominal concentration (ng/ml) | | | |
|  |  |  | High (799.9) | | Low (4.999) | |
| Long term | | Mean | 732. | | 5.081 | |
|  |  | %CV | 7.4 | | 10.9 | |
|  |  | %Bias | -8.4 | | 1.6 | |
| Freeze and thaw | | Mean | 718.6 | | 5.037 | |
|  |  | %CV | 12.3 | | 2.9 | |
|  |  | %Bias | -10.2 | | 0.8 | |
| On bench | | Mean | 716.9 | | 5.370 | |
|  |  | %CV | 5.5 | | 12.7 | |
|  |  | %Bias | -10.4 | | 7.4 | |

All results are mean of n = 6

### Cross validation

No significant differences were found between the samples prepared in human blood and in blood from mice. This indicated that human blood could be used to prepare calibration standards and quality control samples. The results are presented in Table 6.

**Table 6** Cross validation result summary for TK900D

| Species | Human | Mouse | Human | Mouse | Human | Mouse | Human | Mouse |
| --- | --- | --- | --- | --- | --- | --- | --- | --- |
| Nominal conc.(ng/ml) | 800 | 800 | 100 | 100 | 10.0 | 10.0 | 2.00 | 2.00 |
| Mean (n = 6) | 852 | 915 | 96.3 | 104 | 9.15 | 10.1 | 1.92 | 2.16 |
| %CV | 14.1 | 6.8 | 7.7 | 8.6 | 7.0 | 8.5 | 5.1 | 8.3 |
| %Bias | 6.5 | 14.4 | -3.7 | 4.2 | -8.5 | 0.5 | -4.1 | 8.1 |

### Matrix effect

It has been noted that co-eluting, undetected endogenous matrix components may affect the ion intensity of the analyte and metabolite and adversely affect the reproducibility and accuracy of the LC-MS/MS [3]. In order to determine whether this effect (called the matrix effect) is present or not, normal blank human blood from 10 different sources was extracted, dried and reconstituted using solutions of high (800.0 ng/ml) and low (10.01 ng/ml) concentrations of the analyte and at one concentration of the internal standard (100.0 ng/ml). These samples were injected together with samples prepared in the reconstituted solution at the same concentrations, containing no matrix components.

The matrix effect is quantitatively measured by calculating the Internal Standard-Normalized Matrix Factor (IS-MF), which is the Peak Area Ratio in the Presence of Matrix Ions for each blood sample divided by the mean of the Peak Area Ratio in the Absence of Matrix Ions.

A matrix factor (MF) of one signifies no matrix effect, while a value of less than one suggests the suppression of ionization. A value that is greater than one signifies ionization enhancement [4]. An absolute Internal Standard-Normalized MF of one is not required for a reliable analytical assay. However, the variability (% CV) in matrix factors should be less than or equal to 15% to ensure reproducibility of the analysis.

The internal standard normalized matrix factor as calculated for this particular paper showed no significant ion suppression or enhancement at high and low concentrations of NP046. The variability (% CV) was 2.6% and 6.4% at 799.9 ng/ml and 4.999 ng/ml, respectively, which indicates that sample analysis was reproducible.

Conclusion

Robust LC-MS/MS method was developed and validated for the quantification of NP046 in blood, using a very small extraction volume (20 μl).

The reported method offers an advantage of rapid and simple liquid-liquid extraction, together with a short chromatographic run time. This makes the method suitable for the analysis of large sample batches without any loss in instrument performance. The signal-to-noise ratios (S/N) at the pre-set LLOQ value of 2 ng/ml, were 70. The S/N ratio indicates that the method was highly sensitive; even though a small volume of extraction (20 μl) was used. The method was successfully used to evaluate the pharmacokinetic parameters of NP046 in a mouse model.

References

1. Guidance for Industry: Bioanalytical method validation. U.S. Department of health and human services, food and drug administration; 2001, Retrieved: December 10, 2013; from: <http://www.fda.gov/downloads/Drugs/Guidances/ucm070107.pdf>, pp. 1-25.

2. European Medicines Agency: Guideline on bioanalytical method validation. 2012, Retrieved: December 18, 2013; from: <http://www.ema.europa.eu/docs/en_GB/...guideline/.../WC500109686.pdf>, pp. 1-22

3. Matuszewski BK, Constanzer ML, Chavez-Eng CM: Matrix effect in Quantitative LC/MS/MS analyses of biological fluids: a method for determination of finasteride in human plasma at picogram per milliliter concentrations. Anal Chem 1998, 70:882–889.

4. Viswanathan CT, Bansal S, Booth B, DeStefano AJ, Rose MJ, Sailstad J, Shah VP, Skelly JP, Swann PG, Weiner R: Workshop/Conference Report – Quantitative bioanalytical methods validation and implementation: best practices for chromatographic and ligand binding assay. AAPS J 2007, 9:E30–E42.
