# Supplementary material for: Efficacy and pharmacokinetic evaluation of a novel anti-malarial compound (NP046) in a mouse model
Source: Malar J. 2015 Jan 6;14:8. doi: 10.1186/1475-2875-14-8 (PMC4326489; doi:10.1186/1475-2875-14-8)
Supplement: Supplementary file 3 — Additional file 3: Plasma protein binding (PPB) assay procedure. The data provided describes the procedure used for the determination of nonspecific binding of the test drug to the micro-partitioning device followed by the PPB-assay using ultrafiltration and ultracentrifugation methods. (DOCX 21 KB) [file 12936_2014_3683_MOESM3_ESM.docx]

**Plasma protein binding (PPB) assay procedure:**

1. ***Procedure for the determination of nonspecific binding by Ultrafiltration*** (procedure was adopted from Dow, 2002; with a slight modification).

- 0.01 M PBS, pH 7.4, was prepared by dissolving 4 g of NaCl, 0.1 g of KCl, 0.722 g of Na_2_HPO_4_, and 0.12 g of KH_2_PO_4_ with water to a final volume of 500 ml.
- Primary stock solutions of TK900D, TK900E, and NP046 at 100 µg/ml; and secondary stock solutions at 1 µg/ml in methanol were prepared.
- PBS aliquots of 990 µl in 2 ml microfuge tubes in duplicates per concentration of each test compound were prepared, and then 10 µl of the test compound stock solutions were added individually into the PBS aliquots.
- Solutions were vortex mixed and incubated for 20 minutes in a water bath at 37^o^C to allow samples to reach physiological temperature.
- Using a long glass Pasteur pipet, samples were transferred into the upper portion of the micro partition device containing a membrane filter with 30 KDa MWCO (amicon Microcon^®^ Centrifugal Filter Device).
- Centrifuge samples at 14000 g for 20 minutes at 37^o^C.
- The ultrafiltrate were extracted and analysed quantitatively using the validated LC-MS/MS bioanalytical drug assay methods specified in Chapters 8 and 9.
- The %NSB were calculated using the equation:

$NSB (\%) = 100 - \left[\frac{\mathrm{Re}sponse from Ultrafiltrate Extracts}{\mathrm{Re}sponse from Extract of a Pure Solution} \times100\right]$;

where pure solution refers to the extract of unfiltered PBS spiked with the analyte of interest at the same concentration as those PBS samples subjected to ultrafiltration.

1. ***Preparation of plasma for the determination of PPB:***

- Thaw plasma aliquots in a water bath at 22^o^C.
- Centrifuge plasma aliquots at 2000 g for 5 minutes, and decant and pool the supernatant.
- Adjust the pH of the plasma pool to7.4 by adding few grains of either solid NaH_2_PO_4_ (decrease pH) or Na_2_HPO_4_ (increased pH).
- Determine the PPB in the same manner as described for the nonspecific binding, replacing PBS with pooled plasma (see steps 2 to 8).
- Calculate PPB (%) using the same equation described in step 8, by replacing NSB with PPB.

1. ***Procedure for the determination of PPB by Ultracentrifugation*** (procedure was adopted from Barré *et al*., 1985; with a slight modification).

- Thaw plasma aliquots in a water bath at 22^o^C.
- Centrifuge plasma aliquots at 2000 g for 5 minutes, and decant and pool the supernatant.
- Prepare plasma and PBS samples at a high concentration (1000 ng/ml), and low concentration (10 ng/ml) for all the test compounds under investigation. The PBS samples were used to determine the response of the unbound (or free) drug, and were not subjected to ultracentrifugation.
- Plasma samples were incubated at 37^o^C in a water bath for 20minutes to allow samples to reach physiological temperature, and then the samples were aliquoted into BECKMAN Quick-Seal centrifuge tubes (13 ml).
- Ultracentrifuge plasma samples at 2 x 10^5^ g for 6 hours at 37^o^C using BECKMAN L8-70M ultracentrifuge (80Ti).
- The supernatant was extracted, and its response was compared with that of the PBS extract after being analysed by the validated LC-MS/MS bioanalytical drug assay methods stated in chapters 8 and 9.
- PPB% was calculated using the equation:

$$PPB (\%) = \left[(T - F) T\right] \times100$$

Where T = total drug concentration; and F = free drug concentration.
