# Supplementary material for: Efficacy and pharmacokinetic evaluation of a novel anti-malarial compound (NP046) in a mouse model
Source: Malar J. 2015 Jan 6;14:8. doi: 10.1186/1475-2875-14-8 (PMC4326489; doi:10.1186/1475-2875-14-8)
Supplement: Supplementary file 4 — Additional file 4: Summary of the assay method for the determination of kinetic solubility. The data provided describes the assay procedure used for the determination of the kinetic solubility of the test drug. (DOCX 21 KB) [file 12936_2014_3683_MOESM4_ESM.docx]

**Summary of the assay method for the determination kinetic solubility:**

The assay procedure was adopted from Alan Hill (www.physchem.org.uk) and ALERA LABS (www.aleralabs.com/solubility-assays.html) with a slight modification.

- PBS was prepared and its pH adjusted to 2.0 and 7.4
- primary stock solutions of the test compounds and controls (reserpine and hydrocortisone) in DMSO at 10 mM were prepared
- STDs at 220, 100, and 11.0 µM, and aqueous solutions at 200 µM were prepared in triplicate in a clear flat bottom 96-well plate by diluting the primary stock solutions in DMSO and PBS, respectively. Samples were briefly vortex mixed
- samples were incubated for 2 hours while mixing in an incubator with orbital shaker at 200 rpm
- samples were centrifuged at 3500 rpm for 15 minutes at 22^o^C
- the supernatant of the standards were transferred from the clear 96-well plate into an opaque, round bottom 96-well analysis plate, while the aqueous samples were filtered using a vacuum manifold through a multi-screen filter plate (MSRLN 0450, 0.4 µM), into the same opaque, round bottom 96-well plate
- samples were analysed using HPLC-UV method by injecting the STDs in duplicate, and the samples in singlet.
