# Supplementary material for: Efficacy and pharmacokinetic evaluation of a novel anti-malarial compound (NP046) in a mouse model
Source: Malar J. 2015 Jan 6;14:8. doi: 10.1186/1475-2875-14-8 (PMC4326489; doi:10.1186/1475-2875-14-8)
Supplement: Supplementary file 5 — Additional file 5: Summary of the assay method for the determination of microsomal stability. The data provided describes the assay procedure used for the determination of the microsomal stability of the test drug in human and mouse microsomes. (DOCX 24 KB) [file 12936_2014_3683_MOESM5_ESM.docx]

**Summary of the assay method for the determination of microsomal stability:**

The assay method was adopted from Obach, 1999, Giuliano *et al*., 2005, and Naritomi *et al*., 2001, with a slight modification.

1. The following stock solution required for the metabolic stability assessment was prepared:
   - 1. 0.1 M phosphate buffer at pH 7.4
     2. 10 mM NADPH (Nicotine amide adenine dinucleotide phosphate) in 0.1 M phosphate buffer
     3. Working internal standard solution (WIS) of 0.1 µM carbamazepine in acetonitrile (kept ice cold all the way through the process of metabolic stability assessment)
     4. 10 mM of the controls (midazolam, propranolol and MMV390048) and the test compounds (TK900D, TK900E and NP046) in DMSO.
2. The liver microsome (human and mouse) was thawed at 37^o^C, and diluted to 0.4 mg/ml in a preheated (at 37^o^C) 0.1 M phosphate buffer.
3. The primary stock solutions (10 mM) of the controls and test compounds were diluted as follows: first in DMSO to obtain 400 µM solutions followed by dilution in phosphate buffer to 40 µM, and were vortex mixed well by vortexing at each step of dilution.
4. A reaction premix (in triplicate for each control and test compound) were prepared by mixing 15 µl of the 40 µM solution with 530 µl of 0.4 mg/ml microsome mixture in a premix plate, and mixed carefully using a plate shaker. The third well from each replicate of the controls and test compounds were used to make the 3x T60 (-NADPH), i.e., to be incubated without NADPH.
5. The incubation mixtures were prepared by pipetting 90 µl of the premix (step 4) into each of the round holed 96-deep well incubation plates for T = 0, 5, 10, 30, and 60 minutes.
6. 300 µl of the ice cold WIS was added to the T=0 plate and kept at ~ -20^o^C.
7. 10 µl of 10 mM NADPH was added to the reaction mixtures to start the reaction except for the 3x T60 (-NADPH) samples.
8. Plates were covered with a sealing mat and the mixture incubated in a shaking water bath at 37^o^C.
9. 300 µl of the ice cold WIS was added to stop the reaction at each time point and plates were shaken using a plate shaker, followed by placing them at ~ -20^o^C for 30 minutes.
10. Samples were centrifuged at 3500 g for 30 minutes at ~ -20^o^C and the supernatant transferred carefully into a 96-well plate (0.22 µM PVDF, with part no. MSGVN22) multiscreen filter plate without dislodging or disturbing the protein pellet.
11. Samples were filtered using a vacuum filter into an opaque, round bottom 96-well plate, and after covering plates with a sealing mat samples were submitted for an LC-MS/MS analysis.

The intrinsic clearance (ml/min/kg) was calculated using the equation (Obach et al., 1997):

$$CL'_{\mathrm{int}} =\frac{0.693}{in vitro T_{1/2}} \times\frac{mL incubation}{mg microsomes} \times\frac{gm liver weight}{kg body weight} \times\frac{45 mg microsomes}{gm liver}$$
