# Supplementary material for: Efficacy and pharmacokinetic evaluation of a novel anti-malarial compound (NP046) in a mouse model
Source: Malar J. 2015 Jan 6;14:8. doi: 10.1186/1475-2875-14-8 (PMC4326489; doi:10.1186/1475-2875-14-8)
Supplement: Supplementary file 6 — Additional file 6: Caco-2 permeability assay protocol. The data provided describes the assay procedure used for the determination of the permeability of the test drug through a biological membrane. (DOCX 22 KB) [file 12936_2014_3683_MOESM6_ESM.docx]

## Caco-2 permeability assay protocol

### I. Materials required:

- Caco-2 cells
- 24-well polystyrene plates (Sigma)
- 6.5 mm Transwell with 0.4 µm pore polycarbonate membrane insert (Sigma)
- Dulbecco’s modified Eagle`s medium (DMEM) in 10% foetal calf serum (FCS)
- 0.2% Trypsin/1 mM EDTA
- Transport buffer: Hank’s balanced salt solution (HBSS) + 10 mM HEPES + 0.35 g/ml NaHCO_3_, pH 7.4 (1 :100 1 M HEPES in HBSS)
- DMSO
- Low permeability control: Lucifer yellow solution (Sigma)
- High permeability control: atenolol (50%0, propranolol (90%), cimetidine (95%) or terbutaline (73%)
- Test compounds (at 100 µg/ml)
- PBS

### II. Cultivation of Caco-2 cells on culture flasks

- Caco-2 cells were maintained at 37^o^C in DMEM in a humidified atmosphere of 5% CO_2_, and the medium was changed every two days
- Cells were subcultured at 70-80% confluence by splitting them with trypsin

### III. Cultivation of Caco-2 cell monolayers on permeable supports

- Confluent Caco-2 cells were subcultured at passage 30-40
- The desired number of filters were placed in cell culture clusters
- Filters were prewet with 50 µl of the medium for two minutes followed by seeding cells at ~ 20000 cells per insert by dispensing 200 µl of the resuspended cell solution
- The plate was incubated at 37^o^C (not exceeding 16 hours)
- The non-adherent cells were removed by replacing the apical medium with 200 µl of fresh medium. This prevents the risk of multilayer formation. Do not aspirate top of medium for the first nine days.
- The medium was changed on alternate days, first basolaterally and then apically, which contains 200 and 600 µl, respectively.

### IV. Experimental procedure:

Cells were used for experiment between days 14 and 21 post seeding. Donor solutions (including a 100 µg/ml Lucifer yellow) were prepared, and all solutions used in this experiment were prewarmed to 37^o^C. Lucifer yellow and TEER (Transepithelial electrical resistance) were used as indicators for the determination of the monolayer integrity.

#### IVa. Lucifer yellow method:

- Residual medium was removed by rinsing the monolayer with HBSS (do not aspirate)
- 200 µl of 6 mM Lucifer yellow was added in to the apical chambers of the monolayers inserted in a plate containing 600 µl of HBSS
- The plate was placed at 37^o^C with orbital shaking (60 rpm) for 1 hour
- A standard curve was prepared from 0.5 to 50 µM
- HBSS blank containing 1 to 0.5% DMSO were placed in the well
- Aliquots of 200 µl were transferred from the receiver well to a solid black plate
- Aliquots of 50 µl were transferred from the transwell insert to another solid black plate containing 50 µl HBSS with 1 – 0.5% DMSO
- Plates were read in a fluorescent reader (Excitation/Emission wavelength 480/530 nm)
- LY rejection (P_c_) values were calculated.

#### IVb. TEER Method:

- At the end of the growth period plates were equilibrated at room temperature for one hour
- The electrical resistance across the monolayer was measured using an Ohm meter equipped with probes, positioning the probes one inside the filter well and the second into the medium in the growth well
- The electrical resistance for each well were recorded
- The resistance of a blank insert (without cells) was determined to subtract the background value.

#### IVc. Drug transport assay:

- The monolayer was rinsed with HBSS
- Test compounds (200 µl) was added into the apical chambers of the monolayers inserted in a plate containing 600 µl HBSS
- The plate was placed in a water bath with orbital shaker (60 rpm) for 2 hours at 37^o^C
- Fifty micro litters of the solution were removed from the apical and basolateral wells and analysed using LC-MS method.
